# Supplementary material for: Limiting extracellular matrix expansion in diet-induced obese mice reduces cardiac insulin resistance and prevents myocardial remodelling
Source: Mol Metab. 2024 Jun 20;86:101970. doi: 10.1016/j.molmet.2024.101970 (PMC11334943; doi:10.1016/j.molmet.2024.101970)
Supplement: Multimedia component 1 [file mmc1.pdf]

Supplemental Table 1

Supplemental Table 1. mRNA expression was determined by qRT-PCR using primers shown in the table.

Gene	Primer sequence
**TNF-α**	F: TGCCACCTTTTGACAGTGAT
	R: GATTTGAAGCTGGATGCTCT
**IL-1β**	F: TGCCACCTTTTGACAGTGAT
	R: GATTTGAAGCTGGATGCTCT
**IL-6**	F: GAAAAGAGTTGTGCAATGGCAAT
	R: TTGGTAGCATCCATCATTTCTTTG
**IL-10**	F: ACTGGCATGAGGATCAGCAG
	R: CTCCTTGATTTCTGGGCCAT
**BNP**	F: ACAGAAGCTGCTGGAGCTGA
	R: CCGATCCGGTCTATCTTGTG
**β-MHC**	F: TATCGATGACCTGGAGCTGA
	R: AGTATTGACCTTGTCTTCCTC
**18s**	F: GCAATTATTCCCCATGAACG
	R: GGCCTCACTAAACCATCCAA

Supplemental Table 2

Supplemental Table 2. Body weights of mice that were used in various study cohorts. Unpaired student t test was used for statistical analysis. *P<0.05 Chow-fed vs HF-fed; $^{\Delta}$P<0.05 HF-Vehicle vs HF-PEGPH20. Animal numbers (N) may not match those in respective Figures/Tables indicated below, and this is because the sample size for each measurement varied due to differences in animal attrition rates and data variations.

**Figures/Tables**	**Body Weight (g)**	
**Fig 1A-E**	Chow-fed<br>HF-fed	26.2 ± 0.9 (N=12)<br>42.4 ± 1.4*(N=12)
**Fig 1F-I**	HF-fed *MMP9*$^{+/+}$<br>HF-fed *MMP9*$^{-/-}$	42.3 ± 1.5 (N=9)<br>39.8 ± 1.3 (N=6)
**Fig 1J-L**	Vehicle HF<br>PEGPH20 HF	37.9 ± 0.4 (N=4)<br>36.7 ± 0.6 (N=4)
**Table 1/Fig 2-5/Suppl. Fig 1-2**	Lean-Control<br>HF-Vehicle<br>HF-PEGPH20	missing<br>45.7 ± 1.7 (N=11)<br>39.7 ± 1.2$^{\Delta}$(N=15)
**Table 2/Fig 6-9**	HF-Vehicle<br>HF-Pirfenidone	39.8 ± 1.5 (N=10)<br>40.9 ± 1.1 (N=9)

Supplemental Figure 1

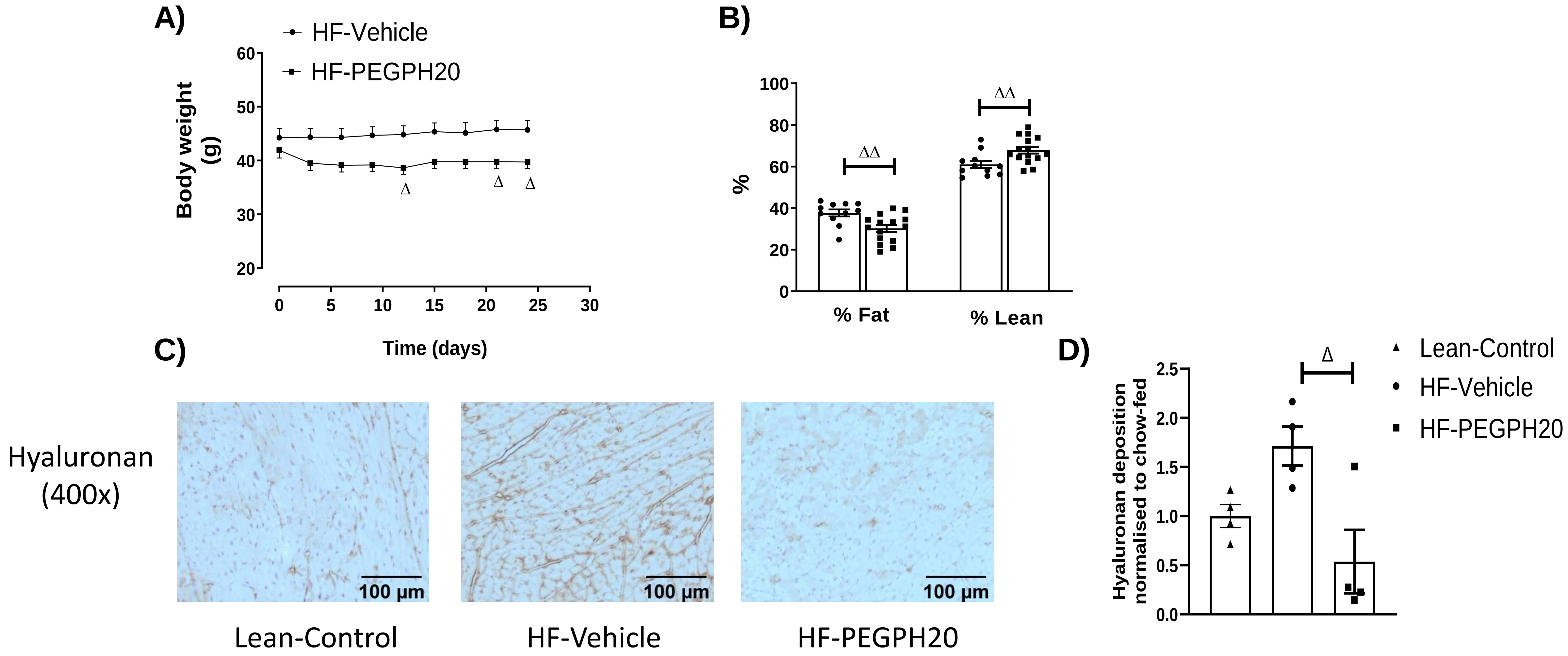

Supplemental Figure 1. PEGPH20 treatment decreased body weight and %fat mass but increased %lean mass in high fat (HF) diet-fed mice. C57BL/6 mice were fed a 60% HF diet for 16 weeks. After 12 weeks of feeding, mice received either vehicle or PEGPH20, once every 3 days for 24 days. (A) Body weight was monitored daily. N=11-15. (B) Body composition was determined after the vehicle/drug treatment. N=11-15. (C) Hyaluronan was detected by immunohistochemistry and quantified by ImageJ in left ventricle sections. Representative images were shown at 400x magnification. N=4. Two-way ANOVA followed by Tukey's method for multiple comparison was used for statistical analysis for Panels A and B, and one-way ANOVA was used for Panel D. $^{\Delta}p<0.05$, $^{\Delta\Delta}p<0.01$ compared with HF-Vehicle. Bar scale 100 µM.

Supplemental Figure 2

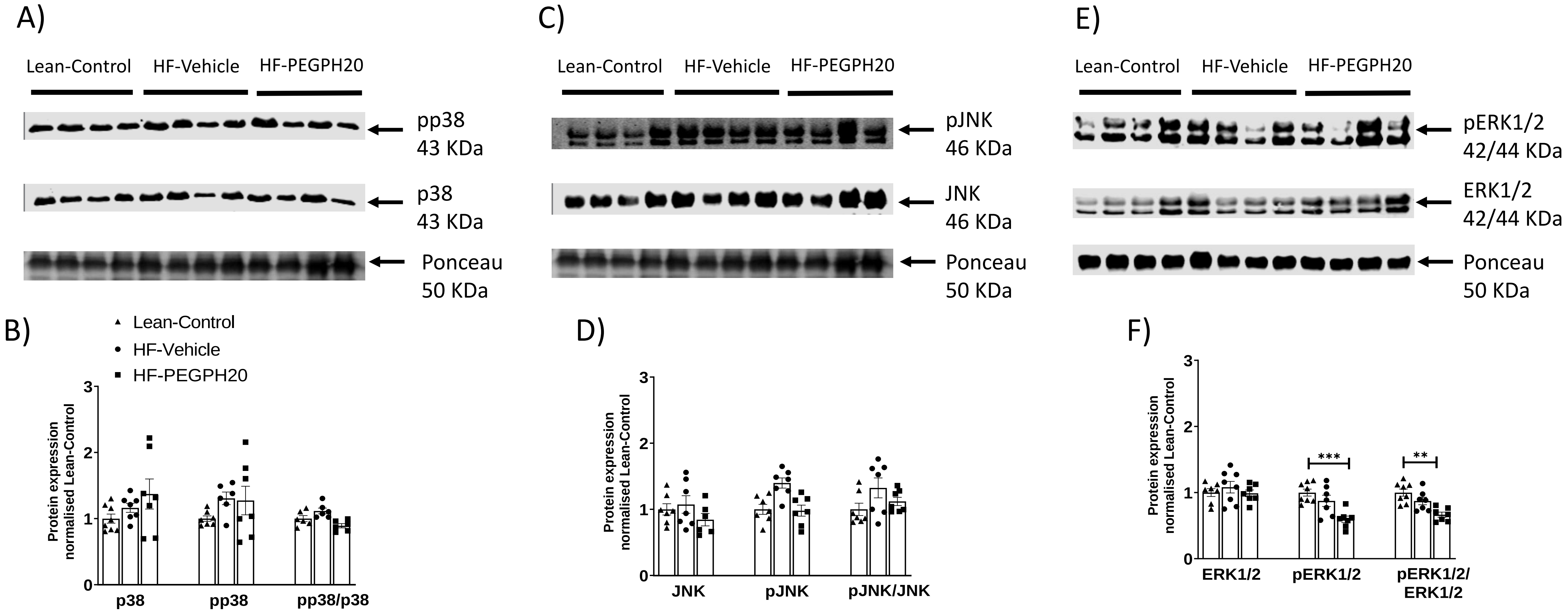

Supplemental Figure 2. MAPK signalling was not altered by high fat (HF) feeding or PEGPH20 treatment, except that pERK1/2 and ratio of pERK1/2 and total ERK1/2 were lower in HF-PEGPH20 mice relative to lean control mice. Protein expression was determined by Western blotting. Representative blots were shown. N=6-8. One-way ANOVA was used for statistical analysis. ***p*<0.01, ****p*<0.005.
